# Telerehabilitation in individuals with severe acquired brain injury: Rationale, study design, and methodology

**DOI:** 10.1097/MD.0000000000013292

**Published:** 2018-12-14

**Authors:** Rocco Salvatore Calabrò, Alessia Bramanti, Martina Garzon, Antonio Celesti, Margherita Russo, Simona Portaro, Antonino Naro, Alfredo Manuli, Paolo Tonin, Placido Bramanti

**Affiliations:** a IRCCS Centro Neurolesi Bonino Pulejo; b IRCCS San Camillo Hospital Foundation, Venice; c University of Messina, Messina; d U.O.C. Neurologia GOM Melacrino-Morelli-Bianchi, Reggio Calabria; e S. Anna Institute and Research in Advanced Neurorehabilitation (RAN), Crotone, Italy.

**Keywords:** healthcare costs, severe acquired brain injury, telerehabilitation, territorial rehabilitative physical treatments

## Abstract

**Background:**

Severe acquired brain injury (SABI) rehabilitation should be as intensive and long as to allow the patients get the best independence and quality of life (QoL), but facing with the rehabilitation socioeconomic burden. Telerehabilitation (TR) could supply frail subjects requiring long-term rehabilitation.

**Methods:**

A multicenter, prospective, parallel design, single-blind trial will be conducted at the IRCCS Neurolesi Bonino Pulejo (Messina, Italy) and IRCCS Hospital San Camillo (Venice, Italy) involving patients suffering from SABI and requiring home motor and cognitive rehabilitation. We will investigate the use of TR, based on advanced Information and Communication Technology (ICT) solutions, taking into account that the supervision of rehabilitation at home will be enriched with the counseling and vital parameters monitoring. The enrolled patients will be balanced for pathology, and randomized in 2 groups, performing TR (G1) or standard rehabilitation training (G2), respectively, according to a pc-generated random assignment. TR will be delivered by means of an advanced video-conferencing system, whereas the patient will be provided with low-cost monitoring devices, able to collect data about his/her health status and QoL. In both the groups each treatment (either cognitive or motor, or both as per patient functional status) will last about 1 hour a day, 5 days/week, for 12 weeks. Two structured telephone interviews will be administered to the patients (when possible) and/or their caregivers, and to all the healthcare professionals involved in the patient management, 1 week after the beginning and at the end of the TR. All the patients will undergo a complete neurological and cognitive examination performed by skilled physicians and psychologists, blindly. Clinical evaluations will be administered blindly, before and after the treatments.

**Results:**

the data of this study should demonstrate that TR is at least non-inferior in comparison with the same amount of usual territorial rehabilitative physical treatments, taking into account patients’ functional recovery, psychological well-being, caregiver burden, and healthcare costs.

**Conclusion:**

data coming from this study could demonstrate the usefulness of TR in facing the rehabilitation socioeconomic burden of managing patients with SABI, so to allow the patients get the best independence and quality of life (QoL).

## 1 Introduction

### 1.1 Background and rationale

Severe acquired brain injury (SABI) is damage to the brain, occurring after birth from traumatic or nontraumatic causes, and often resulting in deterioration of physical, cognitive, and emotional functions.^[1]^ SABI include a variety of acute brain lesions characterized by the occurrence of variably prolonged coma (24 hours), and simultaneous motor, sensory, cognitive, and/or behavioral impairment that causes a certain degree of disability. Congenital, perinatal, or degenerative-progressive brain injuries are excluded from this definition. The most common causes of SABI are traumatic brain injury (TBI), anoxic encephalopathy and ischemic or hemorrhagic stroke. These conditions mostly affect individuals from the fifth decade onwards and represent about 40% of the SABI. Further nontraumatic SABI arises from brain tumors, infections, and toxic-metabolic encephalopathy.^[2]^ Despite the fact that steady progress has been made toward prolonging patients’ survival and several pharmacologic and neuromodulating strategies have been proposed, results on functional recovery of SABI are still scarce.^[1,2]^

Functional recovery following SABI usually reaches its peak at around 6 months, and begins to decline as soon as 1 year after SABI, although rehabilitation may be effective also in the chronic phase. Thus, the increasing use of rehabilitation postdischarge may result in better motor and cognitive recovery. To this end, supervised stroke rehabilitation in the community for up to 1 year was associated with faster recovery and better functional status than with unsupervised therapy.^[1,2]^

Unfortunately, due to healthcare costs, patients are discharged before reaching the desired autonomy.

Providing rehabilitation at home is limited by high costs and rehabilitation provider availability. Thus, the use of home-based, exercise-oriented interventions by means of TR may be decisive. Indeed, TR allows for continuity of service through the entire rehabilitation cycle including assessment, intervention, consultation, and education, and it has recently emerged as an effective tool to provide rehabilitation care to patients early discharged at home, increasing clinical outcomes, by affording early reintegration and positively enhancing QoL.

### 1.2 Objectives

Aim of this project is to determine whether the use of TR in patients affected by SABI leads to greater (or at least equal) improvement in motor and cognitive function, functional communication, independence in self-care and domestic life, when compared with traditional in-home rehabilitation.

We also aimed to report on the presence of adverse events, feasibility and levels of user satisfaction of SABI patients (living at home) and their caregivers, which are associated to TR, monitoring of patient activities, and health status and telecounseling interventions.

Finally, we aim at evaluating the TR system-related cost-effectiveness, identifying the time necessary to amortize the initial investment for the Information and Communication Technology (ICT) equipment and services set up.

## 2 Methods

### 2.1 Trial design

This is a multicentric observational, rater-blinded, active-controlled, parallel-group pilot study to evaluate that TR is at least noninferior in comparison with the same amount of usual territorial rehabilitative physical treatments (UTRT). This study will be conducted at the Rehabilitative Units of IRCCS Centro Neurolesi, Messina (Unit 1) and of IRCCS Hospital San Camillo, Venice (Unit 2). Unit 3 (University of Messina) will help in Health Technology Assessment (HTA) evaluation and big data management. The study protocol was approved by the Ethical and Research Committee of IRCCS Centro Neurolesi “Bonino-Pulejo,” Messina, Italy (ID: 08/2018). The trial is registered under trial gov. (NCT03709875).

The study purpose will be explained to the participants, and patients’ consent will be obtained before the enrollment by the principle investigator.

The study has been funded by the Italian Ministry of Health: WFR: GR-2016-02361306.

### 2.2 Eligibility criteria

In both the clinical Units, we plan to enroll a total 40 stroke patients (20 for each treatment setting) and 40 TBI patients (20 for each condition) requiring home motor and cognitive rehabilitation.

Inclusion criteria are: age range 18 to 65 years; diagnosis of stroke and TBI (less than 1 year), according to neuroradiological and clinical assessments; availability of a home internet connection; the presence of a Montreal Cognitive assessment ≥16/30; the presence of a skilled caregiver.

### 2.3 Exclusion criteria

Exclusion criteria will be: severe cognitive and behavioral impairments, cardiorespiratory instability or other medical illness potentially interfering with the treatment, severe limb spasticity, high-risk of spontaneous fracture, and substance abuse.

### 2.4 Prescreening

Consecutive patients suffering from SABI, admitted to Rehabilitative Units of IRCCS Centro Neurolesi “Bonino-Pulejo,” Messina (Unit 1) and of IRCCS Hospital San Camillo, Venice (Unit 2), and requiring home motor and cognitive rehabilitation, will be evaluated for enrollment in this pilot study 2 weeks before the discharge to their homes.

### 2.5 Randomization of participants

The enrolled patients will be balanced for pathology and randomized in 2 groups, performing TR (G1) or standard rehabilitation training (G2), respectively, according to a simple randomization scheme generated by a software (www.randomization.com).

### 2.6 Study population

All the patients will be divided into 2 groups, performing TR (G1) or standard rehabilitation training (G2). TR will be delivered by means of an advanced video-conferencing system, whereas the patient will be provided with low-cost monitoring devices, able to collect data about his/her health status and quality of life (QoL). In both the groups each treatment (either cognitive or motor, or both as per patient functional status) will last about 1 hour a day, 5 days/week, for 12 weeks. Clinical evaluations will be administered blindly, before and after the treatments. Besides the clinical scales, evaluation regarding burden of care and HTA will be administered by Unit 1 and 3 to contribute to study aims. Besides the analysis of patient and caregiver burden, we will perform an observational study to analyze the patient's suitability to cope with a new system of rehabilitative treatment delivery. Two structured telephone interviews will be administered to the patients (when possible) and/or their caregivers, and to all the healthcare professionals involved in the patient management, 1 week after the beginning and at the end of the TR.

Satisfaction with the program and occurrence of any problem or barrier will be assessed. Longitudinal evaluation will compare baseline scores reported at the first interview with the posttreatment scores.

Besides the general HTA assessment, a second observational study will be specifically focused on Local Health Units (LHU) to verify whether and to what extent the different organizational settings in 2 diverse Italian regions (i.e., Sicily and Veneto) may influence TR feasibility and costs. Initial investments for ICT solutions necessary for TR (videoconferencing system, monitoring equipment, Cloud-based storage and processing solutions) and direct healthcare cost associated with stroke and TBI survivorship will be assessed. One-year direct cost of stroke will be estimated using hospital and LHU electronic records and interview responses of poststroke patients/caregivers. Costs will be reported for 1 year poststroke, and modeled over the lifetime poststroke.

### 2.7 Clinical evaluation

Neurological examination will be performed by 2 skilled physicians, blindly administering the following Clinical scales: Barthel Index (BI),^[3]^ WHO Disability Assessment schedule (WHODAS-12),^[4]^ Modified Ashworth Scale,^[5]^ Fugl-Meyer,^[6]^ and Tinetti^[7]^ scales. Cognitive status will be evaluated by means of Montreal Cognitive assessment (MoCA),^[8]^ and Frontal Assessment Battery.^[9]^ Beck Depression Inventory (BDI),^[10]^ Coping orientation^[11]^ to problem experienced, Psychological General Well-being Index,^[12]^ and Short-Form-36^[13]^ health outcome will be also blindly administered by a skilled psychologist to evaluate the rehabilitative training-related psychological impact. Finally, Perceived Disease Impact Scale (PDIS)^[14]^ and system usability scale, as well as Caregiver Burden Inventory (CBI),^[15]^ will be also administered to either the patients or caregivers. Besides the clinical scales, evaluation regarding burden of care and HTA will be administered by Unit 1 and 3 to contribute to study aims.

### 2.8 Telerehabilitative and standard rehabilitation training treatment and recording systems

The exercises will be delivered to all patients, following a task-oriented paradigm.

(1) *UTRT rehabilitation*: the patients will receive current care and exercises, adjusted in reason of the clinical needs, as usually. Treatments for motor limbs activity will be focused on functional active-assistive and active exercises. Conventional “paper and pencil” training will be used to improve cognitive function.
(2) *TR* will be delivered by means of an advanced video-conferencing system, whereas the patient will be provided with low-cost monitoring devices, able to collect data about his/her health status and QoL. All treatments from remote are based on scheduled videoconferences between the patient's home and the Clinical Units, so that the therapist can always control and modify the exercises. A virtual reality based system, consisting of 2 PC-based workstations, located at the patient's home and at the rehabilitation center, will be used. For the motor treatments the patient has to move the real end effector, following the trajectory of the corresponding virtual task displayed on his computer screen. The speech (mainly lexical based) and cognitive (attention focused) exercises will be delivered from the 2 Research Institutes to the patient's home. During the treatment at home, the patients will use wearable monitoring devices to monitor their status (speed, heart rate, respiratory rate, training load, and single-lead ECG in real-time) and to provide real-time feedback during exercises.

**Figure 1:**
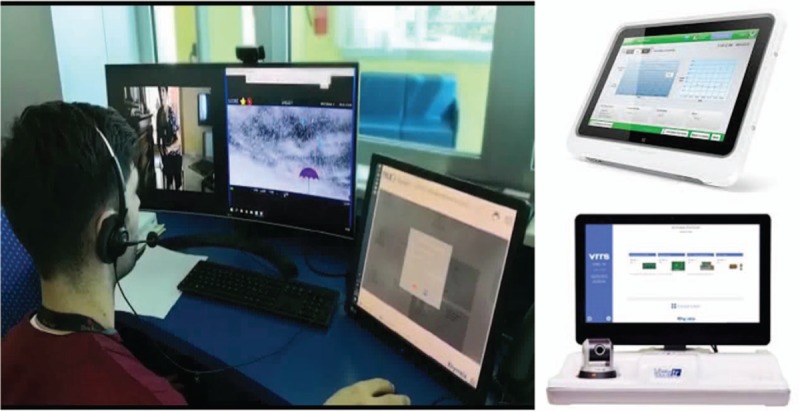
Typical telerehab training by using the work station tele-cockpit. On the right, the VRRS-Tablet (on the top) and the complete home VRRS-TR station (on the bottom).

The training will be delivered by means of the Virtual Reality Rehabilitation System (VRRS). VRRS represents a real clinical and technological innovation that allows TR, integrating different rehabilitation modules (motor, cognitive, linguistic, and orthopedic). The system let a remote patient monitoring at home, thus reducing the costs of health care and the length of hospital stay. Such tool is conceived as a “central HUB” to which it is possible to connect, through a USB, a series of specific devices, that is, VRRS-Tablet. This latter is given to the patients to perform their exercises at home, under the control of a therapist by means of a hub-workstation, called Cockpit (Fig. 1). Patient's activity is controlled remotely from a workstation. The VRRS-Tablet contains different exercises, automatically adapted to the patient's performance, thanks to an optimal intensity training for every cognitive and/or motor condition. In fact, each exercise has a customizable setting efficiency. The VRRS-Tablet is equipped with patient and therapist mode: the former allows performing the exercises and monitoring the data produced, whilst the latter allows monitoring the patient's progress through the platform. These data will be stored in Cloud-based systems for further evaluation and statistical purposes (Unit 3). UTRT will be delivered in each patient's home, according to the local organizational system (i.e., Messina and Venice districts). The data coming from both clinical Units will be stored and analyzed at IRCCS for blind statistical analysis of primary outcome, and for HTA.

### 2.9 Participants timeline

#### 2.9.1 Prescreening visit

Evaluation of inclusion and exclusion criteria. Signature of informed consent.

#### 2.9.2 Visit 1 (baseline)

Medical history.

#### 2.9.3 Visit 2 (at the end of rehabilitative program)

Neurologic and Neuropsychiatric assessment.

### 2.10 Sample size

Based on the pilot samples during study, we calculated a minimal sample size of 60 participants per group in this study, given a power of >80% to detect an interaction in the 2-way repeated measures, an effect size of 0.61, 4 variables, and 2 repeated measurements.

### 2.11 Statistical analyses

The disability level will be the principal outcome, measured by WHODAS-12 (primary outcomes), and upper limp Fugl-Meyer (secondary outcome). The global cognitive status, assessed by MoCA, will be also a primary outcome. According to the data distribution, parametric or nonparametric statistical tests will be used. In particular, the independent Student *t* test or the Mann–Whitney *U* test will be used to compare the 2 groups, whereas the Student *t* test for unpaired samples or the Wilcoxon signed-rank test will be used to compare the data collected at the first evaluation and the end of the treatment (intragroup analysis). Linear correlation between variables will be computed by Pearson's coefficient or nonparametric Spearman's rank correlation coefficient. Statistical significance will be set at bilateral alpha level of 0.05. HTA assessment: Data will be collected by means of structured checklists, harmonized with all the units, to be administered to all involved actors. Any required data pooling, transformation, averaging, statistics and overall data processing will be performed by Unit 1 and 3 for assessing proper HTA indicators. With respect to cost analysis, specific direct, and indirect costs will be retrieved from each experimental study to allow performing Cost-Effectiveness and Cost-Minimization analysis within HTA3, to be possibly compared with corresponding cost analysis within HTA1. TR will be supported by the adoption of advanced ICT systems, necessary to set up videoconferencing between remote therapists and patient at home, monitor patient health status and activities, and store and process data collected during TR sessions. Monitoring systems will be composed by low-cost nonintrusive devices (such as bracelet or others).

### 2.12 Expected outcomes

1. Patients undergoing TR training will have a greater (or at least the same) improvement in nearly all the motor and cognitive scale scoring administered, with reduced disability (as evaluated by BI and WHODAS2).
2. We expect that acceptability and feasibility measures will be significantly higher in the TR group when compared with the conventional treatment, with a concomitant decrease of the caregiver's burden (as per CBI and BDI).
3. From the economic point of view, we expect that TR may be a sustainable, low-cost and effective way to deliver rehabilitative interventions.

## 3 Discussion

Emerging ICT is changing medical and psychological practice by enabling the provision of services across time and distance, yet there are significant concerns about these applications. A recent study by our group have demonstrated that telemedicine can be considered as an important tool in improving health and QoL in the elderly living in nursing homes, and potentially reducing healthcare service access, hospitalization, and costs.^[16]^ It also appears that a telehealth system integrated in a local healthcare service may significantly improve elderly persons’ behavior, and also reduce the caregivers’ burden.^[17]^ Finally, Web-based cognitive rehabilitation has been shown to be useful in improving cognitive performance, besides psychological well-being, in demented individuals living in home care.^[18]^ Specifically, in patients with stroke it has been demonstrated that tele-rehabilitation for motor and higher cortical deficits and in poststroke depression seem to be as or better effective, compared to the “face-to-face” therapies. Although previous data mainly coming from small pilot studies are conflicting, we expect the efficacy of TR is at least noninferior in comparison with the same amount of conventional territorial rehabilitative physical treatments usually delivered to patients suffering from SABI. Exploiting advanced ICT solutions for TR, we also expect a greater improvement in patients’ cognitive and psychological status (as evaluated by specific scales and questionnaires), as well as an improvement in caregivers’ QoL. Taking in account the transfer costs and the time expenditure of conventional rehabilitation, we expect the costs of TR could be inferior in comparison with the costs of UTRT. Moreover, although Chen et al have found no significant differences in abilities of activities of daily living and motor function between conventional and TR, further and more recent studies showed evidence of efficacy in favor of the tele-rehabilitation.^[19–23]^

A recent Cochrane Review found insufficient evidence to reach conclusions about the effectiveness of TR after stroke, as the authors were unable to find any randomized trials that included an evaluation of cost-effectiveness, and to state which intervention approaches was most appropriately adapted to a TR approach.^[17,24–37]^

Our project not only will evaluate the clinical efficacy of TR in a general population (living in 2 different Italian districts), in comparison with conventional neurorehabilitation, but also takes in consideration the cost/efficacy of the system, its feasibility/usability and QoL of the patients and their caregivers.

## 4 Ethics and dissemination

### 4.1 Ethical requirements

The Principal Investigator (PI) will conduct the study according to the current version of Declaration of Helsinki, the Good Clinical Practice guidelines, and the local regulations.

### 4.2 Ethics committee

The PI will obtain ethics committee approval of the protocol informed consent form, and other required study documents before starting the study. After approval, the informed consent will not be altered without the agreement of the relevant ethics committee. It is the responsibility of the PI to ensure that all aspects of institutional review are conducted in accordance with current governmental regulations. Protocol amendments will be subject to the same requirements as the original protocol. A progress report will be submitted to the ethics committee at required intervals and not less than annually. At the completion or termination of the study, the PI will submit a close-out letter to the ethics committee.

### 4.3 Subject information and consent

A written informed consent will be obtained from all study participants, according to local regulations. The Investigator will inform the subject that participation to the study is voluntary and that refusal will not lead to loss of any benefit or prejudice the relationship with the physician in any way. Before enrolment into the study, each subject will receive a full explanation of the nature and purpose of the study from the Investigator. A clear Information Sheet covering important aspects in writing will be given to the subject who will read it and have the opportunity to ask any questions whatsoever. The subject will be given adequate time for consideration before he/she is requested to sign the informed consent in duplicate. One of the original copies of the signed informed consent form will be kept by the Investigator.

### 4.4 Subject confidentiality and data protection

Before any testing under this protocol, the subjects will also provide all authorizations required by local law (D.Lgs. 196/2003). In agreement with the GCPs, each subject will be identified by a code in an unequivocal manner, which will be the identifier of the subject for the duration of the study.

### 4.5 Study time table and duration

– Projected first patient in: December 2018
– Projected number of patients: SABI patients
– Projected last patient visit: June 2020
– Month-duration: 18 months

## Author contributions

**Conceptualization:** Rocco Salvatore Calabrò, Martina Garzon, Antonio Celesti, Paolo Tonin.

**Data curation:** Rocco Salvatore Calabrò, Alessia Bramanti, Margherita Russo, Simona Portaro, Antonino Naro, Alfredo Manuli, Paolo Tonin, Placido Bramanti.

**Formal analysis:** Antonino Naro.

**Funding acquisition:** Rocco Salvatore Calabrò.

**Investigation:** Rocco Salvatore Calabrò, Martina Garzon, Antonio Celesti, Margherita Russo, Simona Portaro, Antonino Naro, Paolo Tonin, Placido Bramanti.

**Methodology:** Rocco Salvatore Calabrò, Alessia Bramanti, Martina Garzon, Antonio Celesti, Simona Portaro, Antonino Naro, Alfredo Manuli, Paolo Tonin, Placido Bramanti.

**Validation:** Simona Portaro, Alfredo Manuli.

**Visualization:** Rocco Salvatore Calabrò, Alessia Bramanti, Paolo Tonin.

**Writing – original draft:** Rocco Salvatore Calabrò, Martina Garzon, Antonio Celesti, Margherita Russo, Simona Portaro, Antonino Naro, Placido Bramanti.

**Writing – review & editing:** Rocco Salvatore Calabrò, Margherita Russo, Simona Portaro, Placido Bramanti.
